# Environmental Altitude and Host Genetics Shape Divergent Microbiota and a Conserved Resistome in Porcine Intestinal Niches

**DOI:** 10.3390/microorganisms14040832

**Published:** 2026-04-06

**Authors:** Renhao Lai, Zhuomacairang Wang, Pengliang Liu, Jiayin Tong, Zulfiqar Ahmed, Richeng Cui, Yiren Gu, Gan Luo

**Affiliations:** 1 College of Animal & Veterinary Sciences, Southwest Minzu University, Chengdu 610041, China; 2 Colleges of Animal Science & Technology, Huazhong Agricultural University, Wuhan 430070, China; 3 Key Laboratory of Qinghai-Tibetan Plateau Animal Genetic Resource Reservation and Utilization, Ministry of Education, Southwest Minzu University, Chengdu 610041, China; 4 NCLBG&G, Faculty of Veterinary and Animal Sciences, University of Poonch, Rawalakot 12350, Pakistan

**Keywords:** antibiotic resistance genes, gut microbiome, high-altitude adaptation, intestinal contents, pig

## Abstract

Environmental stressors and host genetics influence gut microbiota and antimicrobial resistance, but their combined effects across intestinal niches remain poorly unexplored. We conducted a metagenomic analysis of 60 jejunal and cecal samples from 30 native Chinese pigs across three altitudes (500 m, 1400 m, and 3850 m). The aim was to disentangle the interactive impacts of altitude, breed, and intestinal site on microbiome structure and antibiotic resistome dynamics. The cecal microbiota was taxonomically conserved and strongly associated with breed. Conversely, while jejunal communities exhibited structural variations among the sampled cohorts, differences in alpha diversity (Shannon index, *p* < 0.01) appeared to be primarily associated with breed differences rather than an independent altitudinal effect. High-altitude Tibetan pigs showed an enrichment of *Bifidobacterium* and *Pseudomonas*, which may be linked to hypoxia adaptation. Despite a shared core resistome (88 ARG types), the cecum harbored significantly higher ARG abundance than the jejunum within-breed comparisons of Tibetan pigs across altitudes; this revealed stable ARG profiles (*p* > 0.05) suggesting that, although some descriptive differences were observed, the independent effect of altitude weakens when the genetic effect is taken into account. Furthermore, carbohydrate-active enzymes (e.g., CBM13, GH33) correlated positively with ARG abundance. In conclusion, the jejunum appears to act as an environmentally responsive niche, while the cecum exhibits a higher ARG abundance that is closely associated with the host breed.

## 1. Introduction

The gastrointestinal (GI) microbiome is a key factor in regulating host physiology, immune function, nutrient absorption, and metabolic homeostasis in humans and animals [1]. The gut microbiota serves as an essential interface between host genotype and external environmental signals in pigs and directly influences growth performance, disease resistance, and feed efficiency traits of exceptional significance to livestock production systems [2]. While the dietary effects on the microbial community are apparent, increasing attention is being given to how the host’s genetics and environmental factors, especially extreme ones such as hypoxia at altitude, influence the gut ecosystem and its functional repertoire, including the antibiotic resistome [3,4].

The physiological challenges posed by high-altitude environments include hypoxia, cold, and chronic exposure to high UV radiation. These factors profoundly affect host adaptation to the environment, microbial colonization, and community dynamics. Hypoxia in humans and highland mammals, such as Tibetan antelopes and yaks, has been shown to alter the composition of the microbiota in their guts and its metabolic processes. For example, selective growth of *Clostridium*, *Ruminococcus*, and *Oscillospira* has been observed in high-altitude herbivores, which has been interpreted as microbial co-adaptation to energy limitations and oxygen-restricted niches [5,6]. At the same time, recent research on Tibetan pigs demonstrated that hypoxia induces changes in microbial diversity and functional pathways [7]. However, the mechanisms by which altitude alters the ecology of gut microbes across different intestinal locations, and how the host’s genetic background mediates or intensifies these changes, remain poorly understood. This is particularly true for livestock species, where ecological gradients and breed diversity remain largely underexplored.

Equally important is characterizing the resistome, the collective set of antibiotic resistance genes (ARGs) present within the microbiota. Livestock animals are both a source and a carrier of ARGs, and the consequences of such reservoirs have clinical, environmental, and agricultural implications. While considerable research has focused on how anthropogenic activities drive the selection of ARGs, recent publications indicate that these genes are also deeply integrated into the evolutionary and ecological context of host–microbiome interactions [8,9]. Host genetics in determining the baseline resistome, which is independent of direct antibiotic exposure, is increasingly being recognized. Specifically, the changes in the distribution of ARGs in intestinal compartments (foregut (jejunum) and hindgut (cecum)) in response to varying environmental and genetic conditions remain poorly characterized [10]. This high spatial resolution is essential to assess not only the quantitative abundance and distribution of ARGs but also how they functionally couple to microbial metabolism, particularly carbohydrate-active enzymes (CAZymes) that catalyze fermentation and nutrient assimilation [11].

These complicated interactions can be dissected using the Chinese native pig breeds. In geographical and in genetic composition, these breeds occur across a wide range, from low-country Neijiang and Bama Xiang pigs to plateau-dwelling Tibetan pigs. In contrast to commercial breeds, whose development occurs in the same environment, native pigs have evolved under different ecological forces, making them ideal candidates to study genotype–environment interactions in the development of the gut microbiota and resistome [12,13]. Moreover, the intestinal tract is anatomically separated into function-specific units, such as the jejunum and cecum, providing additional resolution for studying niche-specific microbial assembly and resistance gene architecture.

The previous literature has mostly described single species or general environmental impacts, with limited consideration of genetic origin or intestinal biogeography [14,15]. However, the simultaneous effects of altitude, host genetics, and intestinal niche on the gut microbiome and resistome remain largely unexplored. Besides, the stability of the resistome under constant and varying genetic conditions has not been rigorously assessed, an essential test of whether the host or the environment primarily selects ARGs [16].

To overcome these limitations, we undertook a more exhaustive metagenomic survey of 60 intestinal samples (jejunum and cecum) of 30 native Chinese pigs in five breed-altitude groups: Neijiang pig (500 m), Bama Xiang pig (500 m), and Tibetan pig at low (500 m), mid (1400 m), or high (3850 m) altitudes. The purpose of our study was to separate the relative roles of host genetics, altitude, and intestinal site in shaping gut microbial diversity, taxonomic structure, intestinal antibiotic resistome architecture, and intestinal metabolic co-functional networks. Precisely, we sought to (1) describe microbial composition and diversity in different areas of the intestines and altitudes; (2) describe resistome distribution and determine the resistance to environmental changes in genetically preserved pig populations; and (3) understand the functional interaction between ARGs and CAZymes to determine metabolic signatures of resistance spread. The multi-factorial strategy can help gain new knowledge about microbiome-mediated adaptation to extreme conditions and provide a more sophisticated perspective on antibiotic resistance management through genetic and ecological stewardship of livestock environments.

## 2. Materials and Methods

### 2.1. Ethical Considerations

The Animal Care and Use Committee of the Southwest Minzu University (SMU-202501241) approved all animal experiments conducted and adhered to institutional and national ethical standards of animal research.

### 2.2. Experimental Design and Sample Collection

The number of native Chinese pigs to be included in the study was 30, which represented five breed–altitude combinations: Neijiang pig (NJP, 500 m), Bama Xiang pig (BMXP, 500 m), and low-altitude (LTP, 500 m), mid-altitude (MTP, 1400 m), and high-altitude (HTP, 3850 m) Tibetan pigs. Pigs were selected based on breed and altitude, and each group consisted of six biologically independent animals, comprising three males and three females per breed (mixed sex, totaling 15 males and 15 females). All animals were approximately 1 year of age (adult developmental stage). The specific breeds and their corresponding body weight ranges were as follows: Bama miniature pigs (20–30 kg), Neijiang pigs (50–70 kg), Longquan Tibetan pigs (LTP, 25–35 kg), Kangding Tibetan pigs (MTP, 25–35 kg), and Daocheng Tibetan pigs (HTP, 30–40 kg). The animals were sourced from local farms corresponding to their specific altitude groups. All animals were wild-type (non-genetically modified) and had undergone no previous experimental procedures. They were clinically healthy with a normal immune status and had no history of gastrointestinal diseases. As a limitation of this observational study, it must be noted that while the animals were clinically healthy, the environmental conditions (including exact daily diet, rearing environment, and precise age) were not fully standardized across the different altitudes and breeds due to the extensively managed nature of these native herds. Therefore, the current design supports associations rather than definitive causality. Two intestinal content samples (jejunum and cecum) were collected from each pig, yielding a total of 60 samples. At around 1 year of age, pigs were slaughtered, and the intestinal contents of the jejunum and cecum were collected instantly. Samples were put in sterile cryotubes, snap-frozen in liquid nitrogen, and kept at −80 °C until further processing of DNA extraction (Supplementary Table S1).

### 2.3. DNA Extraction and Metagenomic Sequencing

Each intestinal genomic DNA sample, with an equal amount of DNA in the content sample, was extracted using the QIAamp Fast DNA Stool Mini Kit (Qiagen, Hilden, Germany) according to the manufacturer’s instructions. The quality and quantity of the DNA were measured with Qubit 4.0 Fluorometer and NGS^tm^ dsDNA HS Assay Kit (Thermo Fisher Scientific, Waltham, MA, USA). Agarose gel electrophoresis of 1% DNA was used to show DNA integrity. Metagenomic libraries were prepared using the Hyper Prep Kit (KAPA, Roche, Indianapolis, IN, USA) and sequenced on the DNBSEQ-T7 platform (MGI Tech Co., Shenzhen, China) with paired-end reads of 150 bp. Each sample was sequenced to a depth of 6 Gb to provide sufficient coverage for downstream analysis.

### 2.4. Data Processing and Quality Control

Fastp (v0.23.2) was used to trim adapters and filter raw sequencing data quality [17]. Reads with a quality score less than Q20 or overly long N bases were eliminated. The reads were first cleaned, and then the pig reference genome (Sus scrofa 11.1) was mapped using minimap2 (v2.24) to filter out any host contamination [18]. Mapping scores of reads were filtered out as host contamination, and the rest of the non-host reads were applied in further analyses.

### 2.5. Taxonomic Profiling

Taxonomic classification with Kraken2 (v2.1.1) was performed using a custom database comprising bacterial and archaeal genomes from the Genome Taxonomy Database (GTDB) and eukaryotic microbial sequences from NCBI-nt [19]. The confidence level was set to 0.5, and Bracken (v2.6.0) was used to re-estimate species abundances to obtain correct relative abundances [20].

### 2.6. Metagenomic Assembly and Gene Prediction

First reads that had high quality were assembled using MEGAHIT (v1.2.9) using the meta-sensitive preset [21]. Contigs shorter than 300 bp were filtered out. Unmapped reads were repeatedly recombined and reassembled to enhance assembly coverage. The assembled contigs were predicted to have open reading frames (ORFs) with Prodigal (v2.6.3) in metagenomic mode [22]. MMseqs2 (v13.45111) was used to cluster the predicted coding sequences (CDS) across all samples to form a non-redundant gene catalogue with a sequence identity threshold of 95% and an alignment coverage of 90% [23].

### 2.7. Functional Annotation and Gene Abundance

Calculation of gene abundance was performed by mapping the cleaned reads to the non-redundant gene catalogue using minimap2 with the short-read preset (-ax sr). PCR replicates were purged with bamUtil (v1.0.14), and the number of genes was adjusted using Reads Per Kilobase of transcript (RPKM) values to control for sequencing depth and gene length. The gene functional annotation was conducted based on gene prediction sequence alignment to public databases using MMseqs2 (sensitivity set 5.7). To identify antibiotic resistance genes (ARGs), we used the Comprehensive Antibiotic Resistance Database (CARD) and an annotation tool at the CAZy database [24,25].

### 2.8. Statistical Analysis

Indices of alpha diversity (Shannon, Simpson, Chao1, ACE, and Pielou’s evenness) were analyzed using the vegan package in R (v4.1.0). For beta diversity, Bray–Curtis dissimilarity was computed, and Principal Coordinate Analysis (PCoA) was performed to illustrate differences in microbial communities. Comparisons between groups were statistically significant using the Student’s *t*-test or the Kruskal–Wallis test, and multiple comparisons were corrected with the Benjamini–Hochberg procedure. The difference in microbial abundance and community structure was evaluated using Linear Discriminant Analysis Effect Size (LEfSe) [26]. It was concluded that a threshold of *p* < 0.05 was significant unless otherwise noted.

The abundance of ARGs was compared to examine the resistome in various intestinal niches (jejunum vs. cecum) and at different altitudes. To visualize overlap in ARGs, the Venn diagram was created, whereas stacked bar graphs and heatmap analysis were used to evaluate the relative abundance (RPKM) of ARGs. In a comparative study to determine breed-specific effects, Tibetan pigs (low-, mid-, and high-altitude) and other breeds (Neijiang and Bama Xiang) were compared at the genomic level to identify exclusive resistome signatures. Spearman’s rank correlation was used to evaluate the co-occurrence between the top 20 ARGs and the most abundant bacterial genera or top 50 CAZymes. Pairwise Pearson correlations were calculated to assess relationships within bacterial genera and CAZymes. Furthermore, the Mantel test was applied to evaluate the correlation between the ARG distance matrix and specific factors (genera and CAZymes). ‘*’, ‘**’, ‘***’ represent *p* value < 0.05, 0.01, and 0.001, respectively.

## 3. Results

### 3.1. Distinct Alpha Diversity Patterns in the Jejunum and Cecum Driven by Altitude and Host Genetics

#### 3.1.1. Cecal Microbial Diversity Is Primarily Driven by Host Genetics

**Figure 1:**
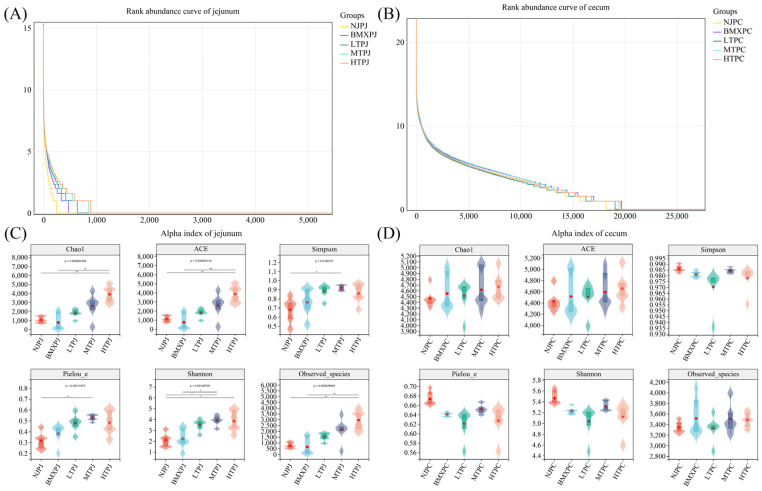
Alpha diversity patterns of jejunal and cecal microbiota in five native Chinese pig groups. (**A**) Rank abundance curves of the jejunal microbiota. (**B**) Rank abundance curves of the cecal microbiota. (**C**) Violin plots of alpha diversity indices (Chao1, ACE, Simpson, Pielou_e, Shannon, and Observed species) in the jejunum. (**D**) Violin plots of alpha diversity indices (Chao1, ACE, Simpson, Pielou_e, Shannon, and Observed species) in the cecum. Data are presented as violin plots overlaid with box plots (*n* = 6 biologically independent samples per group), and statistical significance is indicated by *p* values or asterisks (* *p* < 0.05, ** *p* < 0.01).

Metagenomic sequencing of 60 intestinal samples (jejunum, *n* = 30; cecum, *n* = 30) yielded robust and high-quality data across all groups, with Q20 and Q30 scores consistently exceeding 98% and 96%, respectively (Supplementary Tables S2 and S3). A summary of the taxonomic classification and assembly statistics further highlighted distinct niche-specific characteristics prior to diversity analysis. Specifically, cecal samples consistently harbored a substantially higher and more stable number of classified taxa at the genus and species levels (Supplementary Tables S4 and S5). Furthermore, metagenomic assembly produced larger and more uniform total assembly lengths in the cecum compared to the highly variable jejunal samples (Supplementary Tables S6 and S7). Based on these high-quality genomic profiles, our analysis revealed significant differences in microbial diversity across intestinal locations (Figure 1A–D). Rank abundance curves indicated that the cecum contained a greater number and evenness of microbial communities compared to the jejunum, as indicated by flat slopes and long tails (Figure 1B). The microbial diversity of the cecum was equally high in all groups, and there was a substantial inter-group difference (Figure 1D), which was not consistent with the altitude. Despite this, it is shown that Neijiang pigs had the highest Shannon diversity, while Pielou’s evenness in LTPC, MTPC, and HTPC was similar but lower, suggesting that host genetics is a primary factor associated with cecal microbial structure.

#### 3.1.2. Jejunal Microbial Diversity Is Highly Sensitive to Altitudinal Gradients

Conversely, jejunal communities showed steep rank-abundance curves, indicating dominance by a small number of taxa, especially in low-altitude breeds (Figure 1A). Site-specific patterns were also established using alpha diversity indices. Altitude had a strong positive relationship with jejunal microbial diversity (Figure 1C). Breeders at low altitudes had much lower richness and diversity, and lower Chao1, ACE, Shannon, and observed species indexes (*p* < 0.05). Diversity increased with altitude, and the highest diversity was observed in high-altitude Tibetan pigs (HTPJ) across all diversity measures. These findings imply that the jejunum is highly sensitive to environmental factors, and that microbial diversity is associated with a combination of altitude and host breed.

### 3.2. Divergent Microbial Community Assembly and Altitude-Adaptive Signatures

**Figure 2:**
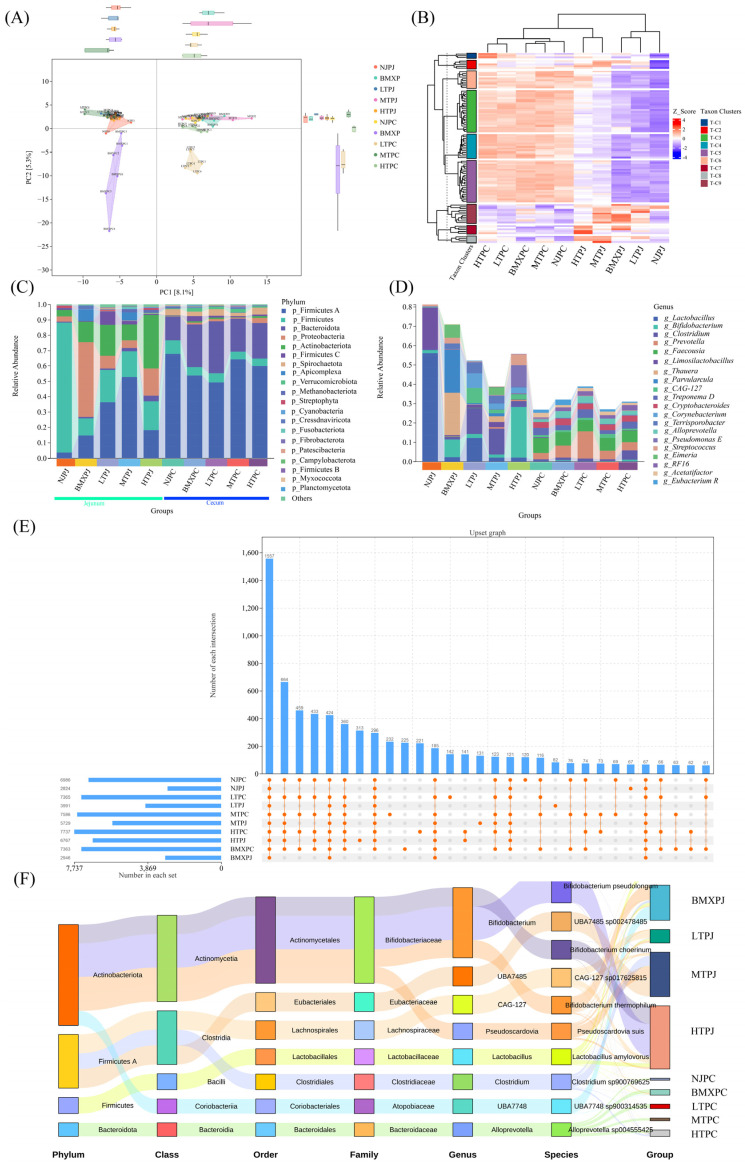
Beta diversity and taxonomic profiling reveal distinct community assembly patterns in the jejunum and cecum across altitudes. (**A**) Principal Coordinate Analysis (PCoA) score plot based on microbial species abundance. (**B**) Heatmap analysis of the top 50 genera clustered by abundance patterns. (**C**) Stacked bar plot showing the relative abundance of bacterial phyla (top 20). (**D**) Stacked bar plot of relative abundance at the genus level (top 20). (**E**) Upset plot illustrating the intersection of microbial taxa (OTUs) across groups. (**F**) Sankey diagram tracing the taxonomic flow from phylum to species level.

The PCoA analysis of beta diversity indicated that jejunal or cecal microbial communities were distinctly separated along PC1, which explained 8.1% of the total variance (Figure 2A). This separation highlights the fundamental ecological difference between the two intestinal niches. The cecum had a stable and preserved microbiome composition of *Firmicutes* and *Bacteroidota* (collectively ≥70% relative abundance; Figure 2C). Core microbiome analysis revealed 459 common genera across all cecal groups (Figure 2E). PCoA clustering revealed a high level of interbreed overlap (Figure 2A), suggesting strong ecological buffering in the hindgut. However, the jejunum showed a high degree of taxonomic polarization and breed-specific dominance (Figure 2B). The Neijiang pigs of low altitude were dominated by *Lactobacillus* and *Limosilactobacillus*, whereas BMXPJ harbored a unique consortium defined by the co-dominance of *Bifidobacterium*, *Thauera*, and *Parvularcula* (Figure 2D). In Tibetan pigs, Low-altitude (LTPJ) and mid-altitude (MTPJ) communities are founded on *Lactobacillus* and *Clostridium*. In contrast, the HTPJ group exhibited a distinctive community feature characterized by a pronounced enrichment and the highest relative abundance of *Actinobacteriota*, a pattern not observed in the other four groups (Figure 2F). From the genus-level community composition, the dominant genera in the HTPJ group were *Bifidobacterium* and *Pseudomonas*. Such reorganization suggests a potential adaptation of facultative anaerobes to hypoxic conditions, though other unmeasured environmental factors cannot be ruled out.

### 3.3. Discriminatory Taxonomic Biomarkers Across Altitudinal Gradients and Intestinal Niches

**Figure 3:**
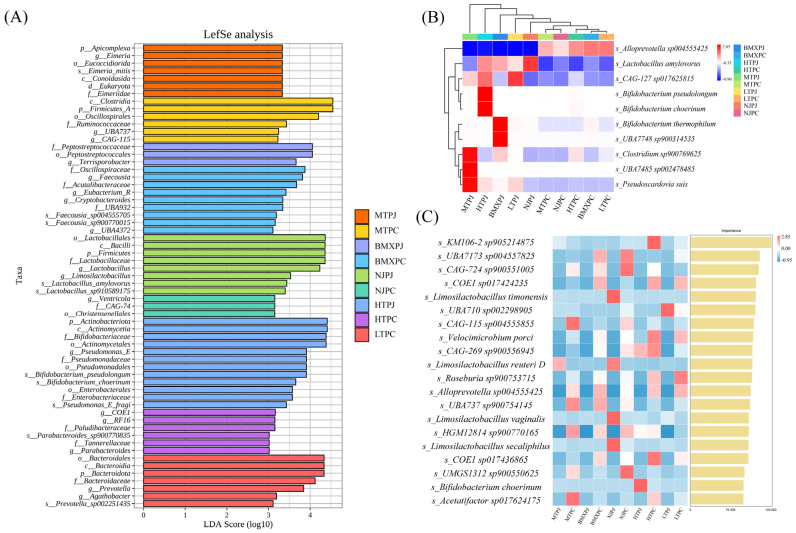
Discriminatory biomarkers and predictive features define breed-specific and altitude-dependent gut microbial signatures. (**A**) Linear Discriminant Analysis Effect Size (LEfSe) identifies differentially abundant taxa (LDA score > 3.0) characterizing each group. The bar length represents the effect size, with colors corresponding to the group where the taxon is significantly enriched. (**B**) Hierarchical clustering heatmap of representative species. (**C**) Random Forest classification analysis identifying the top predictive microbial features. The right bar chart ranks the top 20 microbial species based on their variable importance scores for distinguishing host groups.

LEfSe and Random Forest analyses revealed some important microbial biomarkers that separated the breeds, altitudes, and locations in the intestines (Figure 3A–C). High-altitude Tibetan pigs were enriched in *Paludibacteraceae* and Parabacteroides in the cecum, whereas *Oscillospirales* were enriched in mid-altitude pigs. *Prevotella* and *Bacteroidota* were found in low-altitude Tibetan pigs. Neijiang pigs were found to have *Christensenellales*, and *Faecousia* characterized Bama Xiang pigs. Crucially, Random Forest analysis identified *Alloprevotella* and *Roseburia* as highly predictive features for the cecum. In Neijiang pigs of low altitude, high LDA scores (>3.0) have been predominated by *Lactobacillales taxa* in the jejunum, which are supported by clustering patterns that emphasize *Lactobacillus amylovorus* (Figure 3B). A specific enrichment of the phylum *Apicomplexa* was observed in mid-altitude Tibetan pigs, particularly the protozoan genus *Eimeria*. Co-enrichment of *Bifidobacterium* and *Pseudomonas* was typical of high-altitude Tibetan pigs, and this phenomenon might be associated with the adaptation of facultative anaerobes to high-altitude conditions. The *Limosilactobacillus* and *Bifidobacterium* species were the most predictive in the Random Forest analysis (Figure 3C).

### 3.4. Stratification of the Gut Resistome by Intestinal Niche, Host Genetics, and Altitude

**Figure 4:**
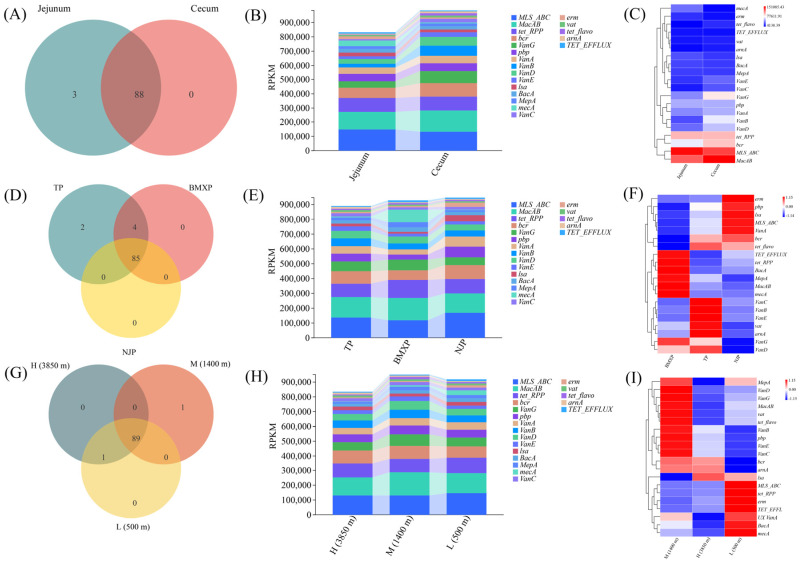
Intestinal niche, host genetics, and altitudinal gradients drive the stratification of the swine gut resistome. (**A**–**C**) Spatial distribution of antibiotic resistance genes (ARGs) between the jejunum and cecum. (**A**) Venn diagram of ARGs between the jejunum and cecum. (**B**) Stacked bar plot of ARG abundance (RPKM). (**C**) Heatmap of ARG abundance (top 20). (**D**–**F**) Breed-dependent resistome profiling across TP, BMXP, and NJP. (**D**) Venn diagram of ARGs across TP, BMXP, and NJP. (**E**) Stacked bar plot of ARG abundance (RPKM). (**F**) Heatmap of ARG abundance (top 20). (**G**–**I**) Impact of altitudinal gradients (High: 3850 m, Mid: 1400 m, Low: 500 m) on resistome assembly. (**G**) Venn diagram of ARGs across altitudes. (**H**) Stacked bar plot of ARG abundance (RPKM). (**I**) Heatmap of ARG abundance (top 20).

ARGs profiling identified a conserved core resistome across intestinal sites, with 88 of 91 ARGs standard in jejunum and cecum (Figure 4A). Nevertheless, the abundance of ARGs was much higher in the cecum (Figure 4B), and the heatmap analysis revealed that multidrug resistance genes such as *MLSABC* and *MacAB* were enriched in this niche (Figure 4C). These genes confer resistance to macrolides, lincosamides, and streptogramins, as well as macrolide efflux, respectively. Because these antibiotic classes are critically important in both veterinary and human medicine for treating severe bacterial infections, their enrichment in the porcine hindgut raises potential public health concerns regarding the accumulation and spread of cross-resistance. Breed-level analysis showed that they exhibited distinct signatures of their resistomes, even though they shared a core of 85 ARGs (Figure 4D–F). Neijiang pigs had the *erm*, *lsa*, and *VanA* genes; Tibetan pigs had vancomycin resistance determinants (*VanB*, *VanC*, and *VanE*); and Bama Xiang pigs had *tetRPP* and *MacAB*. To further clarify the association between altitude and antibiotic resistance genes, we also analyzed the distribution and differential patterns of ARGs in the gut microbiome of Tibetan pigs across different altitudes (Figure 4G,H and Supplementary Figure S2). Specifically, this breed-specific analysis revealed that while Tibetan pigs from high (HTP), middle (MTP), and low (LTP) altitudes shared a highly conserved core resistome (Supplementary Figure S2A), their overall ARG abundance (Supplementary Figure S2B) and the specific enrichment patterns of the top 20 ARGs (Supplementary Figure S2C) were distinctly stratified by elevation. This confirms that altitudinal gradients exert an independent selective pressure on the gut resistome even within a single genetic background. The analysis of altitude showed that ARGs abundance was non-linear, with a peak at the middle altitude (Figure 4H). The hyper-abundance of high-risk ARGs, such as *tet RPP*, *TET_EFFLUX*, *erm*, and several vancomycin-resistance genes, including *vanA* (Figure 4I), might be associated with higher anthropogenic exposure typical of low-altitude agricultural settings. In contrast, these high-risk ARGs were suppressed in mid-altitude and high-altitude populations. While these findings initially suggest that the composition of ARGs could be influenced by altitude and human activities, as detailed in the subsequent section, the independent effect of altitude weakens when the genetic effect is taken into account.

### 3.5. Statistical Validation of Niche-Specific and Host-Driven Resistome Patterns

**Figure 5:**
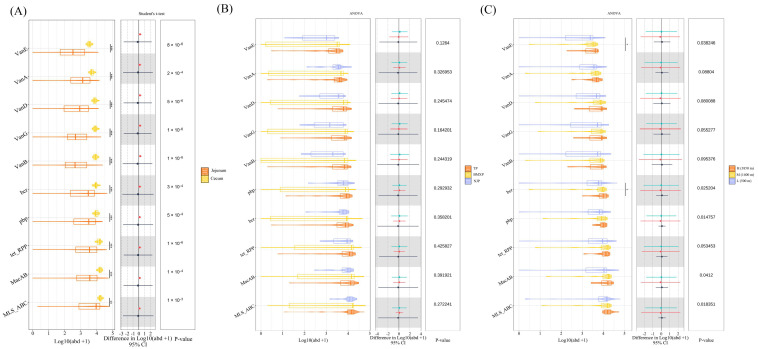
Statistical validation reveals niche-specific enrichment and the dominance of host genotype over altitudinal gradients. (**A**) Differential abundance analysis (Student’s *t*-test) of ARGs between jejunum and cecum samples. (**B**) ANOVA comparison of ARG abundance among three distinct pig breeds (TP, BMXP, NJP) raised in the same low-altitude environment. (**C**) ANOVA comparison across altitudinal gradients (High, Mid, Low) involving mixed pig populations. Box plots represent log-transformed abundance [Log10(abd + 1)], and error bars indicate 95% confidence intervals. Statistical significance is indicated by *p* values or asterisks (* *p* < 0.05, ** *p* < 0.01, and *** *p* < 0.001).

The abundance of several ARGs in the cecum was significantly higher than that in the jejunum including vancomycin (*VanA, VanD, VanE*), β-lactam (*pbp*), and multidrug resistance genes (*bcr, MacAB, MLS_ABC*), as confirmed by statistical testing (*p* < 0.01; Figure 5A). Although multivariate tests indicated breed-specific clustering (Figure 4F), the univariate ANOVA results showed no significant differences in individual ARG abundance across breeds reared under identical conditions (*p* > 0.05; Figure 5B), suggesting modulation of the entire system rather than a selective shift in individual genes. Importantly, while some descriptive differences in ARG abundance were observed across altitudes in the mixed population (Figure 5C), this difference was eliminated after correcting for host genetics among the Tibetan pigs (Supplementary Figure S3). This confirms that some descriptive differences have been observed, but the independent effect of altitude weakens when the genetic effect is taken into account.

### 3.6. Taxonomic Reservoirs and Metabolic Coupling Shape the Gut Resistome

**Figure 6:**
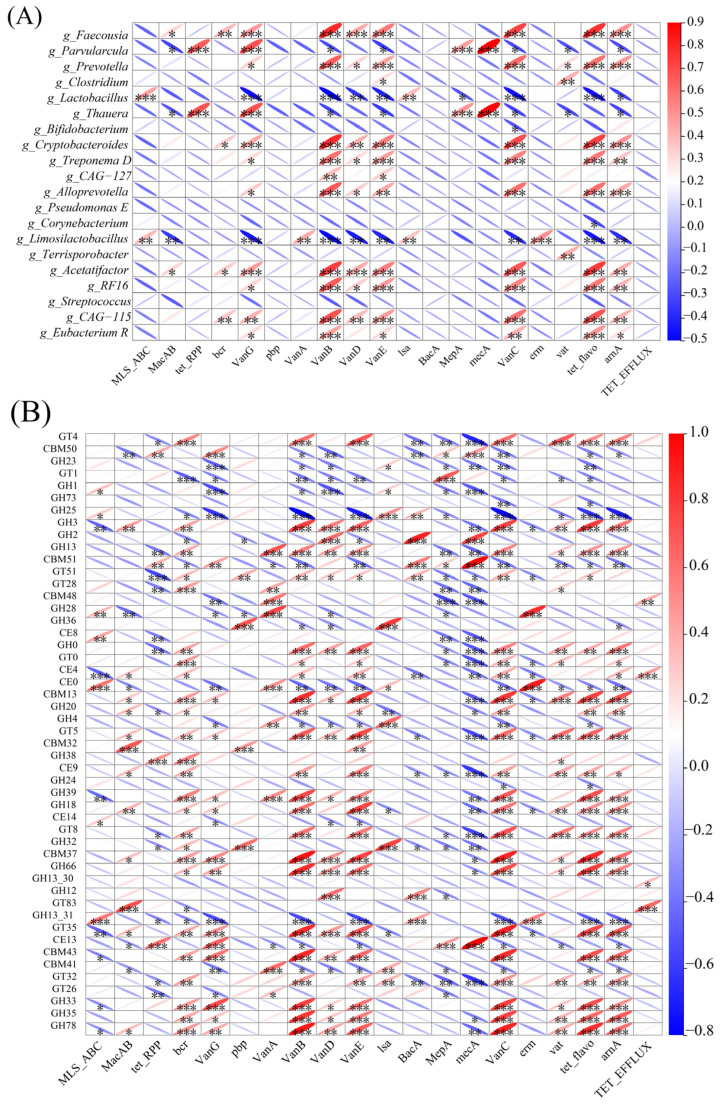
Taxonomic reservoirs and metabolic functional coupling of the gut resistome. (**A**) Co-occurrence analysis between the top 20 most abundant bacterial genera and ARGs (analyzed across all samples). (**B**) Correlation landscape linking the top 50 CAZymes with the top 20 ARGs (analyzed across all samples). In both panels, red and blue ellipses denote positive and negative Spearman correlations, respectively. The color intensity and ellipse shape correspond to the correlation coefficient, and asterisks indicate statistical significance (* *p* < 0.05, ** *p* < 0.01, and *** *p* < 0.001).

**Figure 7:**
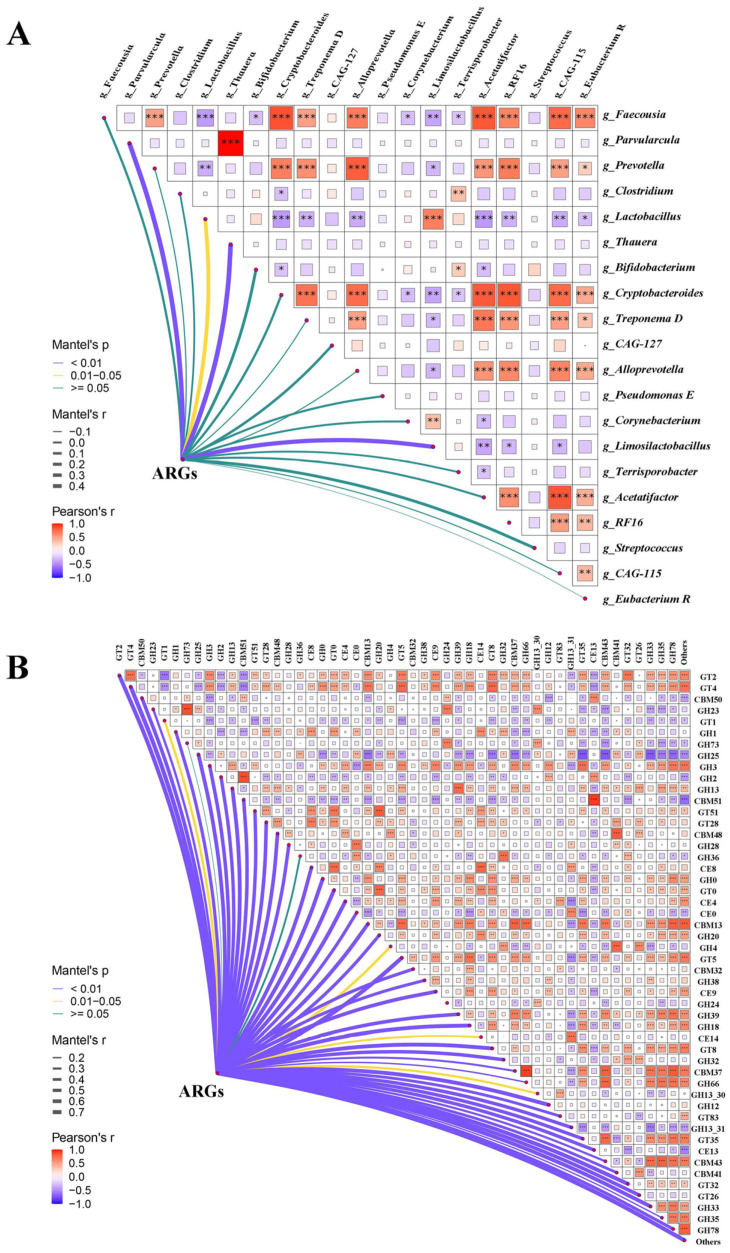
Associations of the gut resistome with bacterial taxonomy and carbohydrate metabolism. (**A**) The correlation landscape between the ARGs (top 20) and the top 20 most abundant bacterial genera (analyzed across all samples). (**B**) The correlation landscape between the ARGs (top 20) and the top 50 CAZymes (analyzed across all samples). In both panels, the triangular heatmaps illustrate pairwise Pearson correlations within the environmental factors (genera in (**A**), CAZymes in (**B**)). The color intensity indicates the direction and strength of the correlation (red for positive, blue for negative), and asterisks denote statistical significance (* *p* < 0.05, ** *p* < 0.01, and *** *p* < 0.001). The connecting lines represent Mantel test analyses linking the ARG matrix to each specific factor. The line width corresponds to the Mantel’s r statistic (correlation strength), and the line color indicates the *p*-value significance level (purple for *p* < 0.01, yellow for 0.01–0.05, and green for *p* ≥ 0.05).

To further understand the relationship between antibiotic resistance genes and microorganisms, we performed a Correlation analysis linking ARGs with the microbiome and CAZymes. The top 20 ARGs (based on relative abundance) were significantly correlated with *Parvularcula*, *Lactobacillus*, *Thauera*, and *Limosilactobacillus* (Figure 6A). Correlation analysis revealed that the major ARG reservoirs included *Prevotella*, *Cryptobacteroides*, *Treponema D*, *Alloprevotella*, and other similar genera, because these genera exhibit significant positive correlations with most ARGs—particularly those conferring resistance to tetracyclines, vancomycin, and macrolides (Figure 6A). *Lactobacillus* and *Limosilactobacillus* were strongly associated with *MLS_ABC*, with a few negative correlations that were both taxa-specific. Notably, the most abundant resistance gene *MLS_ABC* in this study showed significant positive correlations only with *Lactobacillus* and *Limosilactobacillus*. Previous research has established that both *Lactobacillus* and *Limosilactobacillus* are beneficial commensals in the porcine gut microbiota, and here they indeed serve as carriers of highly abundant resistance genes. Conversely, negative correlations were rare. For instance, *Bifidobacterium* showed a significant negative correlation exclusively with *VanC*, while *Corynebacterium* was negatively associated only with *tet_flavo*. The functional coupling analysis showed that in the study of CAZymes and ARGs, there exist strong links (Figure 6B). With the exception of GH36, the top 20 ARGs showed significant correlations with all other CAZymes (Figure 7B). CBM13, CBM37, and GH33 acted as central hubs, exhibiting extensive positive associations with multiple vancomycin (*VanB, VanD, VanE, VanG*) and tetracycline (*tet_flavo*) resistance genes. Some enzymes such as GH38 showed exclusively positive associations, suggesting that bacteria possessing these metabolic capabilities tend to undergo joint selection with ARGs. Negative correlations were also prevalent; for example, GH73 showed negative associations only with *VanC* and *tet_flavo*, indicating that metabolic pathways involving GH73 are likely independent of ARGs. These results indicate potential co-selection in the carbohydrate metabolism and antibiotic resistance in select microbial lineages. However, it must be noted that correlation networks do not demonstrate causality.

## 4. Discussion

In this study, we investigated the jejunal and cecal microbiomes and resistomes of native Chinese pigs across varying altitudinal gradients and genetic backgrounds. Our major findings demonstrate that the porcine gut microbiome exhibits distinct, niche-specific responses: the jejunal microbiome is highly dynamic and primarily associated with environmental factors (e.g., high-altitude hypoxia), whereas the cecal microbiome and its associated resistome are more conserved and predominantly associated with host genetics. The jejunum is an environmentally sensitive and dynamic compartment where hypoxic stress at elevated altitude correlates with significant reorganization of the microbial communities. Conversely, the cecum is taxonomically and functionally stable, serving as a strong, conserved fermentation niche, and the major location of the antibiotic resistome. Biologically, the higher microbial diversity observed in the cecum is crucial for maximizing the degradation of complex, indigestible plant polysaccharides, thereby providing essential short-chain fatty acids (SCFAs) that fulfill a significant portion of the host’s energy requirements. Consistent with this, as *Prevotella* species are well-documented degraders of plant non-starch polysaccharides and play a crucial role in breaking down complex carbohydrates into short-chain fatty acids [27,28], their observed enrichment raises the possibility of a diet-associated adaptation for complex fiber utilization. However, without precise dietary data, this remains a speculative hypothesis. This diverse ecosystem also enhances colonization resistance against opportunistic pathogens, ensuring overall intestinal homeostasis. However, environmental exposures still pose significant health challenges. For instance, the specific enrichment of *Eimeria* observed in mid-altitude Tibetan pigs is likely associated with the harsh living conditions and the extensive foraging habits of free-ranging plateau pigs, where contaminated feed may predispose them to environmental microbes. While *Eimeria* is an intracellular parasite known to cause coccidiosis, its metagenomic detection here lacks corroborating parasitological or clinical data. Therefore, rather than confirming active parasitic infections, this finding merely suggests a potential vulnerability of these extensively managed herds to gastrointestinal exposures.

One striking observation is the breed-driven difference in the alpha diversity of jejunal microbes, where Tibetan pigs maintained higher diversity compared to low-altitude breeds (e.g., NJP and BMXP). This finding contradicts the existing hypothesis of homogeneous suppression of biodiversity by extreme environmental conditions [6,29]. The jejunal communities of low-altitude breeds are characterized by low evenness with *Lactobacillus* and *Limosilactobacillus* dominating as a result of severe competitive exclusion by host-adapted taxa in benign environments [30,31]. In contrast, Tibetan pigs exhibit higher community evenness. Hypoxia at high altitude may further interact with these breed-specific traits to destabilize microbial balance, potentially because it suppresses the fitness of dominant symbionts and decreases colonization resistance, thereby allowing the proliferation of facultative anaerobes such as *Bifidobacterium* and *Pseudomonas*. This trend corresponds to the ecological theory that moderate disturbance improves community evenness by preventing the monopolization of the best competitors [5,32].

The continuous enrichment of *Bifidobacterium* in high-altitude Tibetan pigs across both intestinal locations, as explicitly evidenced by our LEfSe and differential abundance analyses, highlights its imperative role in plateau adaptation. The *Bifidobacterium* species have an extraordinary ability to ferment host mucins under low-oxygen conditions [33], which may exploit the hypoxia-induced rise in mucin secretion as an epithelial defensive response [34]. Increased syntrophic interactions with *Clostridium*, which we observed to be highly correlated in our co-occurrence network models, may also enhance short-chain fatty acid production, further alleviating the energetic limitations imposed by hypoxia and cold stress [35]. This microbial signature is reminiscent of convergent adaptations in other high-altitude mammals, including yaks [6], pikas [36], and Tibetan sheep [37], suggesting that the microbiome might be a fast-evolving extragenomic system that helps the host adapt.

Examination of the resistome shows that host genetics is strongly associated with the baseline antibiotic resistance genes (ARGs) composition, playing a secondary role in native populations that are least exposed to anthropogenic antibiotics. Comparisons of Tibetan pigs across altitudes within the same breed show that the resistome is exceptionally stable, even in the face of radically different environmental conditions, suggesting a strong breed association with the core resistome [38,39]. The breed-specific resistome clustering, despite non-significant univariate differences in individual ARG abundances, suggests that host genotype influences the holistic resistome architecture through selection and maintenance of particular commensal assemblages [40,41]. As a result, the porcine gut resistome does not appear to be a highly vulnerable ecosystem to transient environmental acquisition but rather is inherently connected to the coevolved core microbiome [16].

Integrative correlation studies also elucidate mechanistic links between microbial metabolism and the maintenance of resistance. Specifically, co-occurrences between specific carbohydrate-active enzymes (e.g., CBM13, CBM37, GH33) and ARGs suggest the co-selection of fermentative and resistance phenotypes within polysaccharide-degrading lineages [42]. The correlation of the commensal *Lactobacillus* with the macrolide-lincosamide-streptogramin resistance (*MLS_ABC*) is especially notable, which underscores the likelihood of intrinsic transmission of clinically significant determinants by the probiotic taxa [43]. In high-altitude pigs, which rely on complex plant polysaccharides, the selection of improved CAZyme repertoires can facilitate maintaining or increasing the components of the resistome [44].

Despite the fact that this study introduces new knowledge regarding the dynamics of niche-specific microbiome and resistome in native pigs, there are a number of limitations that must be recognized. First, the sample size (*n* = 6 per group) is relatively small and may not be sufficient to detect interactions that are harder to detect or taxa that are less common, a frequent limitation in animal metagenomic studies. Second, daily ration, antibiotic history, housing, and exact rearing conditions were not completely standardized across altitudes and breeds due to the extensively managed nature of these herds, which may confound other factors affecting microbial composition independently of hypoxia or genetics. Consequently, our current experimental design is observational, and the findings support associations rather than definitive causality. Third, the lack of data on mobile genetic elements, horizontal gene transfer, absolute copy numbers, and precise antibiotic exposure history limits our ability to definitively prove the existence of a genetically anchored resistome reservoir. Furthermore, the correlation networks utilized in this study only indicate statistical co-occurrence and do not demonstrate true biological causality. Additionally, a cross-sectional design does not allow investigation of how temporal dynamics, and shotgun metagenomics, despite its thoroughness, can bias detection of low-abundance or novel ARGs due to limited sequencing depth and database biases.

In future studies, larger longitudinal cohorts employing controlled dietary interventions are required to disentangle intermediary factors and the temporal dynamics of microbiome-resistome relationships. Multi-omics (e.g., metatranscriptomics, metabolomics, and host genomics) integration will clarify the mechanisms underlying the functional changes observed in adaptations and the propagation of resistance. Investigating the potential of mobile genetic elements and horizontal gene transfer in native breeds may help explain the risks of resistome dissemination.

## 5. Conclusions

In conclusion, this study reveals that the porcine gut microbiome and resistome are associated with distinct, niche-specific ecological forces: the jejunum acts as an environmentally fragile compartment responsive to altitudinal hypoxic stress, whereas the cecum serves as a more conserved hindgut environment with higher ARG abundance. This research uncovers that high-altitude adaptation is facilitated by the enrichment of specific taxa (e.g., *Bifidobacterium* and *Pseudomonas*) in the jejunum. Regarding the resistome, some descriptive differences have been observed across altitudes, but the independent effect of altitude weakens when the genetic effect is taken into account, highlighting a stronger association with host genetics. Furthermore, CAZyme–ARG co-occurrences highlight the potential link between microbial metabolism and resistance maintenance. Ultimately, these findings emphasize the critical need to consider both intestinal biogeography and host genetics in livestock management. By leveraging breed-specific microbial signatures, future agricultural strategies can implement precision breeding or probiotic interventions to enhance environmental resilience in native breeds. This approach provides a sustainable, green solution for optimizing livestock production while simultaneously mitigating the risk of antimicrobial resistance dissemination.

## Data Availability

The original contributions presented in this study are included in the article/Supplementary. Further inquiries can be directed to the corresponding authors.

## Supplementary Materials

The following supporting information can be downloaded at: https://www.mdpi.com/article/10.3390/microorganisms14040832/s1, Figure S1: Geographical distribution of sampling sites for the five pig breeds in this study; Figure S2: Altitudinal gradients drive the stratification of Tibetan pigs gut resistome; Figure S3: Controlled comparison within the Tibetan pig lineage negates altitudinal significance; Table S1: The group name and description table for this study; Table S2: Summary of metagenomic sequencing data statistics for the jejunum samples; Table S3: Summary of metagenomic sequencing data statistics for the cecum samples; Table S4: Statistics of taxonomic classification of metagenomic reads from the jejunal samples; Table S5: Statistics of taxonomic classification of metagenomic reads from the cecum samples; Table S6: Overview of assembly quality and sequence characteristics for jejunal samples; Table S7: Overview of assembly quality and sequence characteristics for cecum samples.
