# Supplementary material for: Environmental Altitude and Host Genetics Shape Divergent Microbiota and a Conserved Resistome in Porcine Intestinal Niches
Source: Microorganisms. 2026 Apr 6;14(4):832. doi: 10.3390/microorganisms14040832 (PMC13118436; doi:10.3390/microorganisms14040832)
Supplement: Supplementary file 1 [file microorganisms-14-00832-s001.zip › microorganisms-4217400-supplementary.pdf]

Environmental Altitude and Host Genetics Shape Divergent Microbiota and a Conserved Resistome in Porcine Intestinal Niches

Renhao Lai [1,2,†], Zhuomacairang Wang [1,†], Pengliang Liu [1,3], Jiayin Tong [1], Zulfiqar Ahmed [4], Richeng Cui [1], Yiren Gu [1,3,*] and Gan Luo [1,3,*]

[1] College of Animal & Veterinary Sciences, Southwest Minzu University, Chengdu 610041, China; lrh19980312@163.com (R.L.); 250905012001@stu.swun.edu.cn (Z.W.); pengliangliu1995@163.com (P.L.); 202430903020@stu.swun.edu.cn (J.T.); r_c_choi@163.com (R.C.)

[2] Colleges of Animal Science & Technology, Huazhong Agricultural University, Wuhan 430070, China

[3] Key Laboratory of Qinghai-Tibetan Plateau Animal Genetic Resource Reservation and Utilization, Ministry of Education, Southwest Minzu University, Chengdu 610041, China

[4] NCLBG&G, Faculty of Veterinary and Animal Sciences, University of Poonch, Rawalakot 12350, Pakistan; zulfiqarahmed@upr.edu.pk

* Correspondence: guyiren1128@163.com (Y.G.); 80300306@swun.edu.cn (G.L.)

† These authors contributed equally to this work.

Captions

Figure S1. Geographical distribution of sampling sites for the five pig breeds in this study.
Figure S2. Altitudinal gradients drive the stratification of Tibetan pigs gut resistome.
Figure S3. Controlled comparison within the Tibetan pig lineage negates altitudinal significance.
Table S1 The group name and description table for this study
Table S2 Summary of metagenomic sequencing data statistics for the jejunum samples
Table S3 Summary of metagenomic sequencing data statistics for the cecum samples
Table S4 Statistics of taxonomic classification of metagenomic reads from the jejunal samples
Table S5 Statistics of taxonomic classification of metagenomic reads from the cecum samples
Table S6 Overview of assembly quality and sequence characteristics for jejunal samples
Table S7 Overview of assembly quality and sequence characteristics for cecum samples

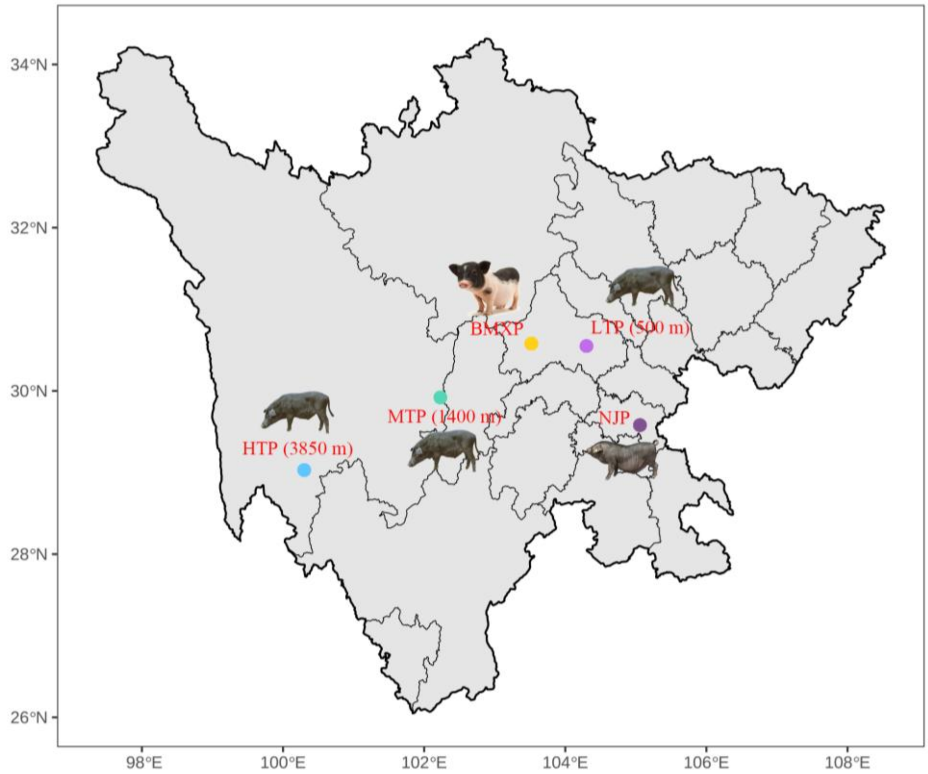

Figure S1. Geographical distribution of sampling sites for the five pig breeds in this study. The colored dots indicate the specific sampling locations in Sichuan Province, China. The abbreviations and corresponding altitudes are as follows: HTP, Daocheng Tibetan pig (High-altitude, 3850 m); MTP, Kangding Tibetan pig (Mid-altitude, 1400 m); LTP, Longquan Tibetan pig (Low-altitude, 500 m); BMXP, Bama Xiang pig (500 m); and NJP, Neijiang pig (500 m).

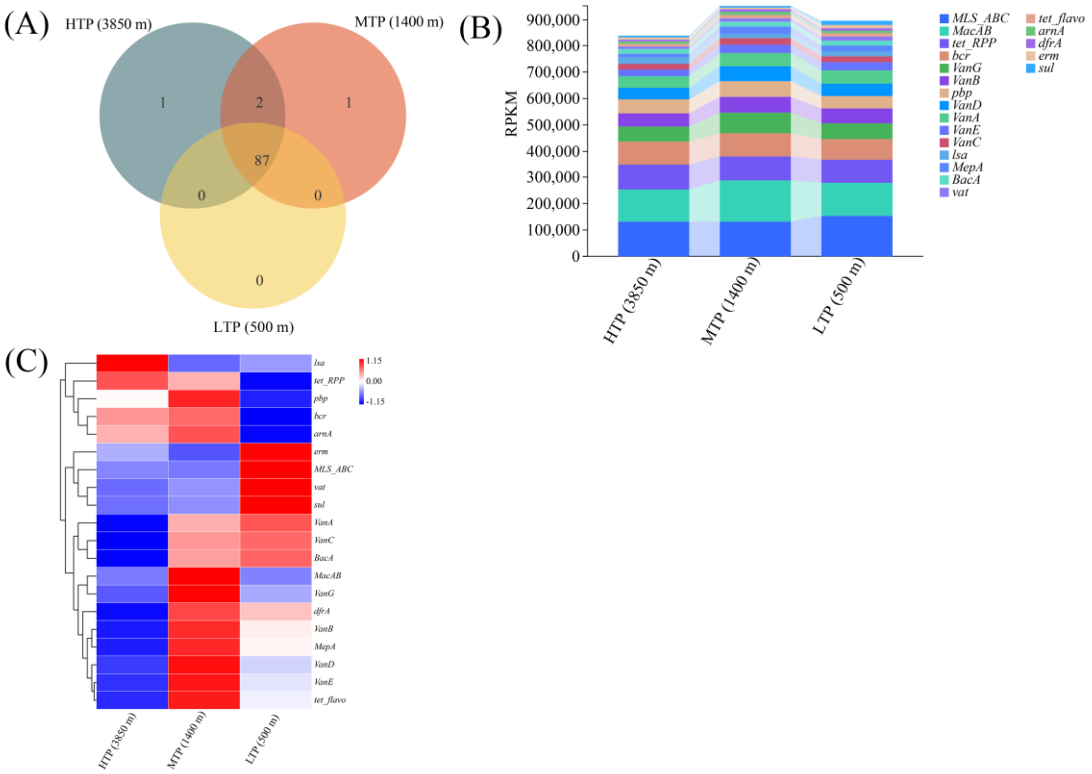

Figure S2. altitudinal gradients drive the stratification of Tibetan pigs gut resistome. A, Venn diagram of ARGs across HTP, MTP, and LTP. B, Stacked bar plot of ARGs abundance (RPKM). C, Heatmap of ARGs abundance(top 20).

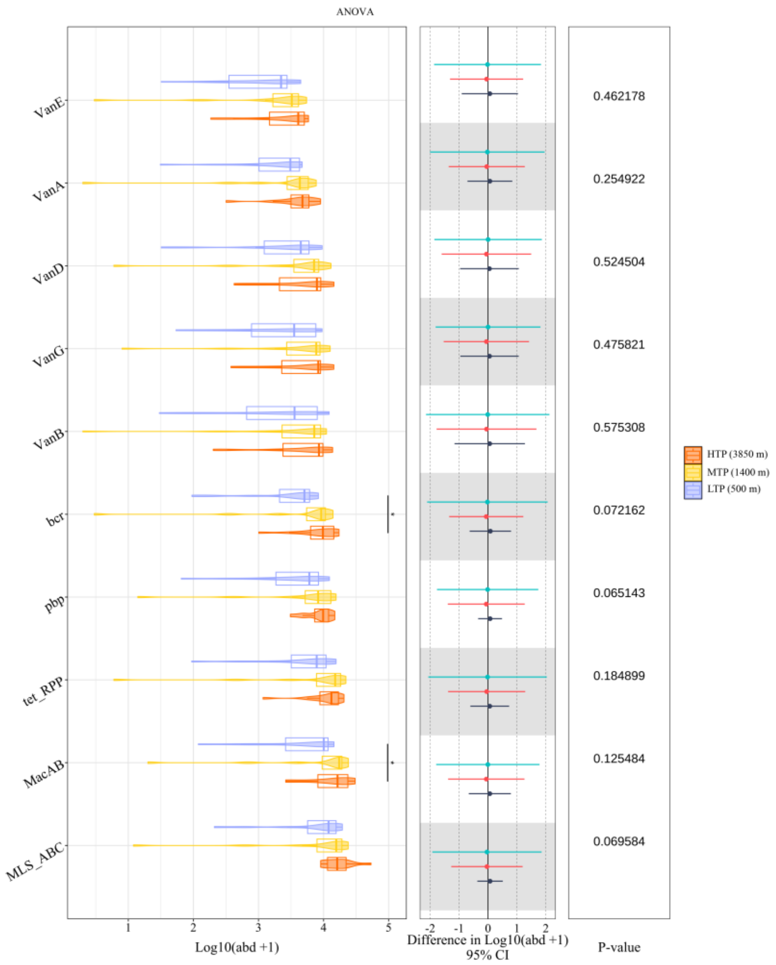

Figure S3. Controlled comparison within the Tibetan pig lineage negates altitudinal significance. Differential abundance analysis (ANOVA) of key ARGs exclusively within Tibetan pig populations across an altitudinal gradient (High: 3850 m, Mid: 1400 m, Low: 500 m).

Table S1 The group name and description table for this study

Abbreviation occurrence location	Groups Abbreviation	Description	Sample size
**Figure 1A and C; Figure 2; Figure 3**	NJPJ	Neijiang Pig Jejunum Contents	6
	BMXPJ	Bama Xiang Pig Jejunum Contents	6
	LTPJ	Low-altitude Tibetan Pig Jejunum Contents	6
	MTPJ	Mid-altitude Tibetan Pig Jejunum Contents	6
	HTPJ	High-altitude Tibetan Pig Jejunum Contents	6
**Figure 1B and D; Figure 2; Figure 3**	NJPC	Neijiang Pig Cecum Contents	6
	BMXPC	Bama Xiang Pig Cecum Contents	6
	LTPC	Low-altitude Tibetan Pig Cecum Contents	6
	MTPC	Mid-altitude Tibetan Pig Cecum Contents	6
	HTPC	High-altitude Tibetan Pig Cecum Contents	6
**Figure 4A, B, and C; Figure 5A**	Jejunum	Neijiang, Bama Xiang,Low-altitude Tibetan, Mid-altitude Tibetan, and High-altitude Tibetan pig Jejunum Contents	30
	Cecum	Neijiang, Bama Xiang,Low-altitude Tibetan, Mid-altitude Tibetan, and High-altitude Tibetan pig Cecum Contents	30
**Figure 4D, E, and F; Figure 5B**	NJP	Neijiang Pig Jejunum and Cecum Contents	12
	BMXP	Bama Xiang Pig Jejunum and Cecum Contents	12

	TP	Low-altitude Tibetan, Mid-altitude Tibetan, High-altitude Tibetan pig Jejunum and Cecum Contents	36
**Figure 4G, H, and I; Figure 5C**	L	Neijiang, Bama Xiang, Low-altitude Tibetan pig Jejunum and Cecum Contents	36
	M	Mid-altitude Tibetan pig Jejunum and Cecum Contents	12
	H	High-altitude Tibetan pig Jejunum and Cecum Contents	12
**Figure S2 and Figure S3**	LTP	Low-altitude Tibetan pig Jejunum and Cecum Contents	12
	MTP	Mid-altitude Tibetan pig Jejunum and Cecum Contents	12
	HTP	High-altitude Tibetan pig Jejunum and Cecum Contents	12

Table S2 Summary of metagenomic sequencing data statistics for the jejunum samples

Sample ID	Raw Reads	Raw Data(bp)	N (%)	GC (%)	Q20 (%)	Q30 (%)
NJPJ1	38573178	5785976700	0.0003	43.31	99.43	97.76
NJPJ2	49149554	7372433100	0	39.57	99.45	97.9
NJPJ3	43767918	6565187700	0.0002	36.46	99.5	98
NJPJ4	45485068	6822760200	0.0002	37.15	99.47	97.88
NJPJ5	45568338	6835250700	0.0003	37.39	99.45	97.78
NJPJ6	45064414	6759662100	0.0002	38.87	99.48	97.92
BMXPJ1	46145492	6921823800	0.0002	43.76	99.28	97.24
BMXPJ2	37662636	5649395400	0.0003	40.83	99.42	97.74
BMXPJ3	53230230	7984534500	0.0002	44.52	99.33	97.49
BMXPJ4	64671418	9700712700	0.0001	42.18	99.29	97.31
BMXPJ5	49233186	7384977900	0.0003	49.85	99.42	97.9
BMXPJ6	45101886	6765282900	0.0003	47.08	99.42	97.86
LTPJ1	38074596	5711189400	0.0003	39.33	99.37	97.62
LTPJ2	38223752	5733562800	0.0003	42.26	99.37	97.63
LTPJ3	52193790	7829068500	0.0001	41.93	99.23	97.07
LTPJ4	42563410	6384511500	0.0002	32.31	99.39	97.6
LTPJ5	51013070	7651960500	0	45.41	99.32	97.53
LTPJ6	42520218	6378032700	0.0002	51.81	99.26	97.24
MTPJ1	40206576	6030986400	0.0003	50.57	99.26	97.27
MTPJ2	43666216	6549932400	0.0002	37.54	99.34	97.41
MTPJ3	60958414	9143762100	0.0001	36.32	99.21	96.96
MTPJ4	41005548	6150832200	0.0002	39.77	99.39	97.66
MTPJ5	48360226	7254033900	0.0003	45.42	99.34	97.63
MTPJ6	52953464	7943019600	0	42.14	99.2	97.05
HTPJ1	53117834	7967675100	0	47.28	99.04	96.52
HTPJ2	48218280	7232742000	0.0001	49.48	98.93	96.06
HTPJ3	42911786	6436767900	0.0002	53.51	98.93	96.12
HTPJ4	44032164	6604824600	0.0003	50.1	99.24	97.14
HTPJ5	41429696	6214454400	0.0002	48.73	98.99	96.31
HTPJ6	45120586	6768087900	0.0003	50.44	99.15	96.87

Table S3 Summary of metagenomic sequencing data statistics for the cecum samples

Sample ID	Raw Reads	Raw Data(bp)	N (%)	GC (%)	Q20 (%)	Q30 (%)
NJPC1	45556054	6833408100	0.0002	45.1	99.33	97.4
NJPC2	42406624	6360993600	0.0002	44.47	99.32	97.35
NJPC3	40725226	6108783900	0.0002	44.57	99.37	97.54
NJPC4	42809358	6421403700	0.0002	44.53	99.4	97.67
NJPC5	46181306	6927195900	0	42.84	99.23	97.1
NJPC6	45962790	6894418500	0	41.38	99.22	97.04
BMXPC1	42371352	6355702800	0.0002	44.45	99.34	97.43
BMXPC2	41986106	6297915900	0.0002	43.08	99.25	97.15
BMXPC3	39783454	5967518100	0.0002	45.97	99.31	97.32
BMXPC4	38420100	5763015000	0.0002	46.15	99.27	97.18
BMXPC5	42842214	6426332100	0.0002	45.07	99.24	97.06
BMXPC6	44497052	6674557800	0.0002	46.25	99.25	97.1
LTPC1	42723678	6408551700	0.0002	43.21	99.13	96.68
LTPC2	36529092	5479363800	0.0003	43.77	99.39	97.6
LTPC3	44328956	6649343400	0.0002	46.16	99.31	97.35
LTPC4	44902528	6735379200	0.0002	45.27	99.33	97.39
LTPC5	40666986	6100047900	0.0002	44.15	99.38	97.56
LTPC6	44595426	6689313900	0.0002	44.21	99.38	97.56
MTPC1	43340656	6501098400	0.0002	46.07	99.31	97.3
MTPC2	43692884	6553932600	0.0002	45.31	99.35	97.48
MTPC3	43448600	6517290000	0.0002	45.25	99.31	97.33
MTPC4	44798096	6719714400	0.0001	44.59	99.29	97.27
MTPC5	37538498	5630774700	0.0003	45.56	99.33	97.39
MTPC6	42584142	6387621300	0.0002	45.02	99.38	97.59
HTPC1	43044016	6456602400	0.0002	43.59	99.34	97.42
HTPC2	40871274	6130691100	0.0002	41.22	99.29	97.24
HTPC3	45768122	6865218300	0.0002	46.09	99.27	97.19
HTPC4	47767104	7165065600	0.0002	45.8	99.3	97.31
HTPC5	42609060	6391359000	0.0002	43.18	99.35	97.47
HTPC6	43296030	6494404500	0.0002	41.95	99.32	97.33

Table S4 Statistics of taxonomic classification of metagenomic reads from the jejunal samples

Sample ID	domain	phylum	class	order	family	genus	species	unclassified
NJPJ1	110	28	23	158	741	1674	14819122	4414592
NJPJ2	10	310	305	351	3262	9098	17671170	6328145
NJPJ3	351	78	41	189	1311	3016	17684710	4170586
NJPJ4	34	28	51	139	767	1702	18589895	4097783
NJPJ5	910	19	55	199	1332	2511	18817630	3930998
NJPJ6	3	45	253	220	1720	4339	18192385	4281213
BMXPJ1	249	35	59	175	1232	2588	10820741	11976692
BMXPJ2	45	30	50	210	1782	3947	5284260	4782465
BMXPJ3	3	0	39	48	347	219	79769	271840
BMXPJ4	19	0	33	6	29	43	62987	55060
BMXPJ5	30	2	66	51	158	408	261217	91148
BMXPJ6	21	4	21	20	58	164	172861	144812
LTPJ1	4	16	27	83	416	664	2825512	1477908
LTPJ2	12	13	11	101	1087	2087	1246033	1038727
LTPJ3	28	27	44	121	1001	2517	269381	198664
LTPJ4	165	59	53	129	1704	3262	7672605	12835177
LTPJ5	7	26	140	579	1884	5356	17220415	7204353
LTPJ6	125	72	195	261	1990	8469	13778547	4590735
MTPJ1	122	56	1929	170	7968	8705	4758930	14910816
MTPJ2	0	79	96	373	3767	15983	7026496	13876871
MTPJ3	19	146	180	678	15116	66893	5193195	22717796
MTPJ4	51	114	147	409	3863	13505	9053198	8530708
MTPJ5	15	20	36	153	1551	2898	691977	883120
MTPJ6	24	3	46	38	124	100	38583	54847
HTPJ1	64	121	118	252	2743	11076	15094202	11418390
HTPJ2	78	68	104	322	2308	7592	14414316	9655333
HTPJ3	155	190	28	310	2088	7231	12278015	9155838
HTPJ4	90	263	146	310	3809	17357	10622009	11290055
HTPJ5	136	172	148	340	2919	15168	10454421	10213326
HTPJ6	114	109	342	449	3431	15540	10372672	11929568

Table S5 Statistics of taxonomic classification of metagenomic reads from the cecum samples

Sample ID	domain	phylum	class	order	family	genus	species	unclassified
NJPC1	24	25	51	155	2359	11621	9014960	11392953
NJPC2	43	64	53	157	2115	12020	8651581	10707529
NJPC3	51	137	80	220	2043	12322	7048208	10975364
NJPC4	11	37	106	135	1999	12569	8372179	12152451
NJPC5	27	65	90	161	2220	12585	9961560	12502957
NJPC6	8	83	50	319	2221	11996	10978365	11644063
BMXPC1	23	119	69	227	1442	14071	6317291	12910069
BMXPC2	22	51	36	362	1451	13610	4385705	11625848
BMXPC3	43	44	117	199	3135	12210	7187193	11505868
BMXPC4	11	85	64	109	1681	12546	6874457	9850775
BMXPC5	143	34	30	234	1551	12355	6701134	14130421
BMXPC6	15	38	30	236	1631	12180	7155444	13162913
LTPC1	18	69	101	492	1951	13396	7232098	12774611
LTPC2	20	36	109	79	2074	12417	7131388	9556572
LTPC3	113	74	36	439	3022	17723	7667456	12249458
LTPC4	25	63	65	588	1829	11139	9128939	12818098
LTPC5	22	27	66	291	1929	12963	7722639	12021880
LTPC6	349	61	75	382	2194	14426	8043278	14205752
MTPC1	526	151	69	118	1834	12392	7573450	13764454
MTPC2	30	52	202	348	1837	14013	6797467	15007790
MTPC3	73	88	275	418	1526	14665	6292905	15223094
MTPC4	54	75	105	393	2272	16517	6249654	13288102
MTPC5	5	111	521	395	2584	15218	5516281	12712485
MTPC6	46	84	126	359	2232	17155	6367373	12859660
HTPC1	145	50	64	520	2270	12430	6381696	15017936
HTPC2	87	70	95	665	2745	13931	7843246	12553542
HTPC3	52	67	37	409	2753	14860	8081807	14719493
HTPC4	505	199	42	306	2796	14274	7784440	16031294
HTPC5	623	98	51	377	2234	14081	6320004	14924047
HTPC6	39	60	84	803	2375	13355	6750139	14855886

Table S6 Overview of assembly quality and sequence characteristics for jejunal samples

Sample ID	Total seq number	N20	N50	N90	Total Length (bp)	GC number
NJPJ1	32342	13653	2592	513	45656896	20150959
NJPJ2	40400	18400	2272	460	50240948	21355401
NJPJ3	34800	10373	1920	489	43768991	15886417
NJPJ4	26655	14000	3160	514	39660047	15729415
NJPJ5	31421	10921	1596	442	33815830	13635289
NJPJ6	28368	16630	3326	562	46215954	21136422
BMXPJ1	85913	17619	2778	501	121361006	52367098
BMXPJ2	74910	12954	2323	479	97094053	40253469
BMXPJ3	3473	1059	616	407	2190697	1025321
BMXPJ4	2021	1200	717	430	1401704	559223
BMXPJ5	2440	3882	1780	487	2856381	1684951
BMXPJ6	1577	1171	614	403	1000297	478005
LTPJ1	43551	8669	2038	480	53945624	16038231
LTPJ2	31057	9036	1793	472	36795752	13476964
LTPJ3	7562	5003	1356	481	8208034	3666713
LTPJ4	118963	6319	1346	419	117886212	38775434
LTPJ5	73135	19405	3184	502	106986531	47882460
LTPJ6	82729	12955	2368	521	115636689	62961750
MTPJ1	74710	15419	2235	505	100671803	46497300
MTPJ2	189936	10980	1889	469	228251026	93945005
MTPJ3	207761	8944	1898	458	244500246	104361237
MTPJ4	193139	6441	1501	459	210229586	82545649
MTPJ5	25263	5115	1364	456	26234936	8652349
MTPJ6	1510	997	638	419	959897	390139
HTPJ1	130306	9487	1657	456	146399365	60924715
HTPJ2	76029	13768	1981	470	92589997	39223377
HTPJ3	60484	16279	2215	476	77483733	35763774
HTPJ4	317940	6709	1396	464	342892839	167392692
HTPJ5	223430	8921	1773	476	265049055	120392225
HTPJ6	215940	9014	1642	461	244707986	112568202

Table S7 Overview of assembly quality and sequence characteristics for cecum samples

Sample ID	Total seq number	N20	N50	N90	Total Length (bp)	GC number
NJPC1	409911	5134	1268	466	424979866	195389172
NJPC2	423971	4516	1173	458	420637974	189109589
NJPC3	382119	5693	1354	470	409540259	186238974
NJPC4	450331	3886	1092	454	430405181	192889199
NJPC5	432248	4966	1164	459	430781977	185166993
NJPC6	347112	10238	1627	481	407399869	170864016
BMXPC1	406070	5103	1240	469	420201675	188378485
BMXPC2	400961	3314	1042	457	375317164	162048109
BMXPC3	428361	4834	1240	464	437584947	202848755
BMXPC4	353114	5153	1275	471	369936167	166887666
BMXPC5	432127	5907	1353	470	462566442	205050090
BMXPC6	458561	5096	1216	463	467217069	215513177
LTPC1	411627	4712	1183	461	412639954	177664944
LTPC2	346136	4463	1116	458	337901521	149589115
LTPC3	396861	4459	1147	460	392358979	177120644
LTPC4	480214	3834	1150	447	460694652	207248345
LTPC5	396119	4049	1131	456	384810454	167547617
LTPC6	504704	4056	1152	460	496914888	219916172
MTPC1	442784	7110	1426	478	491898786	228040609
MTPC2	462157	6079	1375	470	498318399	226207581
MTPC3	503963	4510	1135	460	496741909	224136169
MTPC4	464879	3887	1171	461	459928187	206890358
MTPC5	380768	5134	1272	466	395549589	182807689
MTPC6	419978	3981	1151	459	412209509	186947028
HTPC1	452538	4622	1253	469	467605280	202825033
HTPC2	273838	7578	1567	478	313652452	127881096
HTPC3	477191	4773	1201	461	481692594	221282260
HTPC4	463091	5923	1398	467	499596321	220608852
HTPC5	495646	3739	1139	458	482040173	206512830
HTPC6	440064	5381	1389	467	471515376	198597289
